# Cementitious materials as promising radiative coolers for solar cells

**DOI:** 10.1016/j.isci.2022.105320

**Published:** 2022-10-13

**Authors:** Matteo Cagnoni, Alberto Tibaldi, Jorge S. Dolado, Federica Cappelluti

**Affiliations:** 1 Department of Electronics and Telecommunications, Politecnico di Torino, Corso Duca degli Abruzzi 24, Torino 10129, Italy; 2 Istituto di Elettronica e di Ingegneria dell’Informazione e delle Telecomunicazioni, Consiglio Nazionale delle Ricerche c/o Politecnico di Torino, Corso Duca degli Abruzzi 24, Torino 10129, Italy; 3 Centro de Física de Materiales, CSIC-UPV/EHU, Paseo Manuel de Lardizabal 5, San Sebastián 20018, Spain; 4 Donostia International Physics Center, Paseo Manuel de Lardizabal 4, San Sebastián 20018, Spain

**Keywords:** Applied sciences, Engineering, Solar terrestrial physics

## Abstract

Nowadays, radiative coolers are extensively investigated for the thermal management of solar cells with the aim of improving their performance and lifetime. Current solutions rely on meta-materials with scarce elements or complex fabrication processes, or organic polymers possibly affected by UV degradation. Here, the potential of innovative cement-based solutions as a more sustainable and cost-effective alternative is reported. By combining chemical kinetics, molecular mechanics and electromagnetic simulations, it is shown that the most common cements, *i.e.*, Portland cements, can be equipped with excellent radiative cooling properties, which might enable a reduction of the operating temperature of solar cells by up to 20 K, with outstanding efficiency and lifetime gains. This study represents a first step toward the realization of a novel class of energy-efficient, economically viable and robust radiative coolers, based on cheap and available cementitious materials.

## Highlights

• A multi-scale approach predicts dielectric properties of cementitious materials
• Cementitious slabs can emit thermal radiation in the atmospheric window
• Low-cost cementitious materials can be effective radiative coolers for solar cells
• Cement-based radiative cooling could significantly extend solar cells lifetime

## Graphical abstract

**Figure:**
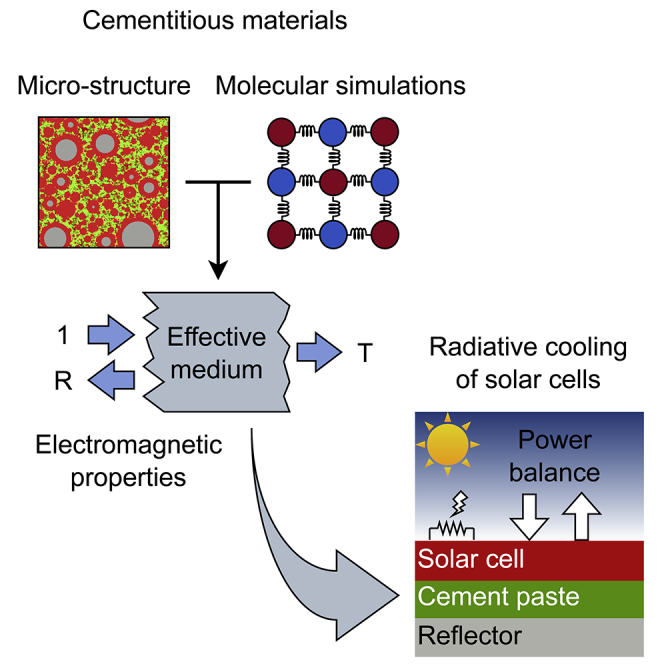

## Introduction

Reducing the operating temperature is a key challenge in solar cells technology. A lower temperature not only increases the power conversion efficiency, by about 0.5%/K in silicon-based devices (Skoplaki and Palyvos, 2009), but also extends the system lifetime, by roughly 2×/10K (Dupré et al., 2017).

Motivated by the possibility of achieving significant gains in performance, researchers have proposed several cooling solutions over the years which are based on diverse concepts (Chandrasekar et al., 2015). Among these, radiative cooling has been attracting much attention lately, not only for the thermal management of solar cells (Li et al., 2017,^2021^; Perrakis et al., 2020,^2021^; Safi and Munday, 2015; Wang et al., 2020), but also for application in buildings (Hossain and Gu, 2016; Li and Fan, 2019; Zhao et al., 2019). This technology stands out thanks to its remarkable potential in terms of energetic efficiency, economical viability, environmental friendliness, and reliability, fostered by its completely passive nature, effectiveness, systemic simplicity and absence of moving parts.

Radiative coolers are bodies designed to strongly emit thermal radiation within the atmosphere transparency window (AW) between 8 and 13μm (see Figure S1) (Catalanotti et al., 1975). Radiation ejected through this channel dodges the bounce-back effect of the atmosphere and reaches outer space without returning to the sender. This uncompensated energy removal reduces the temperature of radiative coolers. Moreover, if strict spectral requirements are fulfilled, sub-ambient temperature can be reached even under direct sunlight, as experimentally proven only recently (Raman et al., 2014).

Thanks to these characteristics, radiative coolers can act as effective heat sinks when thermally coupled to a warming body, such as a solar cell. Indeed, the excess heat generated within the cell on sunlight absorption is going to flow toward the colder radiative cooler. Then, the latter is going to permanently remove it from the system in the form of thermal radiation through the atmospheric window. Remarkably, this process can reduce the operating temperature of silicon-based devices by up to 18.5K (Zhu et al., 2014), roughly leading to a 9% efficiency gain (Skoplaki and Palyvos, 2009) and 360% of lifetime (Dupré et al., 2017).

Different kinds of radiative coolers were discovered in the last few years (Hossain and Gu, 2016). The most common ones are meta-materials made of vertically stacked thin films (Li et al., 2017; Raman et al., 2014; Kecebas et al., 2017), or thick layers with a micro-patterned surface (Perrakis et al., 2021; Zhu et al., 2014; Hossain et al., 2015; Kong et al., 2019). Yet, it is unclear whether these technologies are adaptable to large-scale manufacturing, because of their reliance on scarce materials such as Ag or Hf (EuChemS, 2021), or complex deposition and patterning methods. To overcome these issues, organic materials such as hierarchical porous polymers have been proposed as a low cost alternative (Carlosena et al., 2021; Wang et al., 2021b, a; Li et al., 2019; Mandal et al., 2018), but their use might be jeopardized by UV degradation (Zhao et al., 2019). This impasse is forcing researchers into a trade-off between performance, cost, and reliability, and calls for prompt action to identify alternative classes of radiative coolers capable of fulfilling all these requirements simultaneously.

Faced with this challenge, we have considered several options and identified (meta-)concretes as a very promising class of cheap and scalable (meta-)materials. Conventional concrete is made by gluing together aggregates such as sand or gravel with a cement paste (binder) (Allen and Iano, 2019). These aggregates can be replaced with more “exotic” inclusions to form *meta*-concrete, a concrete-like meta-material that can be equipped with unconventional properties (Mitchell et al., 2014). A first hint at the possible application of these materials as radiative coolers is provided by their multi-scale porous structure (Dolado and Van Breugel, 2011), which strongly resembles the one of the aforementioned hierarchical porous polymers. Furthermore, there exist many recipes for the cement paste and many possible choices for the aggregates, which lead to concretes with very different chemistry and micro-structure (Aïtcin, 2000; Bensted and Barnes, 2002; Bohnet and Ullmann, 2003). As a matter of fact, these materials form an extremely broad class, which can be used not only in buildings (Gagg, 2014), but also in clinical applications such as bone prostheses (Kenny and Buggy, 2003) and tooth restoration (Chadwick and Evans, 2007). This characteristic provides researchers with many knobs to tune properties. Finally, concretes are already being investigated in the context of buildings as a structural material equipped with radiative cooling capacities (European Commission, 2021), with recent experiments confirming their strong thermal emissivity in the atmospheric window (Lu et al., 2021) and high reflectance at sunlight wavelengths (Levinson and Akbari, 2002).

Encouraged by these observations, we have transferred for the first time with this work the idea of cement- and concrete-based radiative coolers from buildings to solar cells. In particular, we have investigated the suitability of ordinary Portland cements (OPC) (Taylor, 2004), which are the most commonly used type of binder, for their thermal management. Remarkably, we have discovered that they can be equipped with dielectric properties suitable for the thermal management of solar cells and potentially capable of providing outstanding gains in performance. These findings may represent a major breakthrough in radiative cooling research, because the main elements found in these cements, such as Ca, Si, O, and H, are among the cheapest and most available on Earth (EuChemS, 2021). At the same time, the stability and reliability of cement- and concrete-based solutions is something that we experience every day. With this article, we demonstrate that cements and concretes can also be equipped with the properties needed for the effective radiative cooling of solar cells and can become the ultimate radiative coolers, capable of fulfilling performance, cost and reliability requirements at the same time. These findings call for further research aimed at realizing an ultimate photovoltaic system design and fabrication protocol.

**Figure 1:**
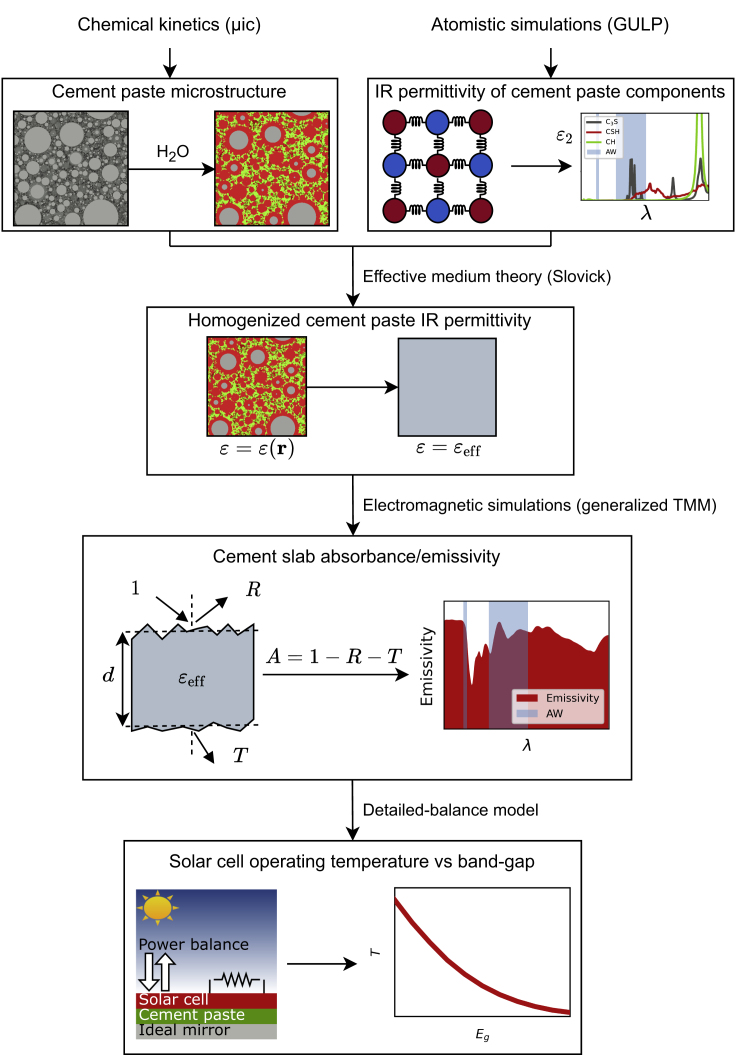
Workflow for the assessment of cement-based radiative coolers for the thermal management of solar cells

To reach these conclusions, we have defined a multi-scale interdisciplinary simulation workflow that calculates the cement paste electromagnetic properties from scratch and uses them in a power balance model to estimate the solar cell temperature reduction driven by its coupling to the cement-based radiative cooler. The essentials of the workflow are depicted in Figure 1.

First, several cement micro-structures are generated by modeling the cement paste formation with methods from chemical kinetics. At the same time, the IR dielectric properties of the basic components of the heterogeneous cement paste are calculated by atomistic simulations. Next, these micro-structural and dielectric data are combined into a suitable effective medium theory to convert the space-dependent dielectric function into an equivalent homogeneous dielectric function. Then, this is plugged into an electromagnetic simulator to calculate the cement slab emissivity. Finally, this slab is “attached” to the solar cell and the operating temperature of the resulting device is calculated by the detailed balance model.

More information on each of these methods and the corresponding findings is provided in the results and discussion section, with additional details in the STAR methods section and the supplemental information.

## Results and discussion

**Figure 2:**
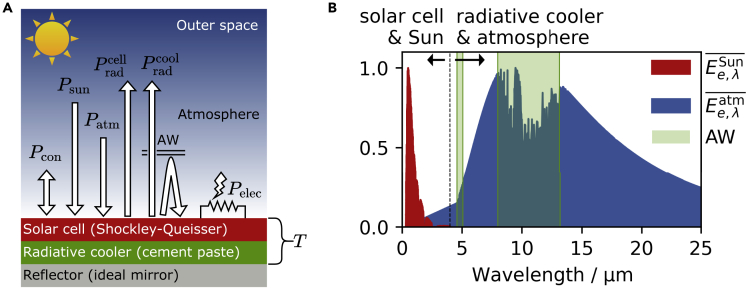
Illustration of the detailed balance model employed (A) Planar structure used to assess the suitability of a radiative cooler for the thermal management of solar cells. The power density terms representing the channels through which the device, the environment and the end-user load exchange energy are also depicted, together with the atmosphere radiation shielding effect outside of its transparency window (AW). (B) Comparison between the main electromagnetic spectra involved in the operation of a solar cell equipped with a radiative cooler. $\bar{E_{e,\lambda }^{\text{Sun}}}$ is the normalized Sun spectral irradiance (AM1.5g), $\bar{E_{e,\lambda }^{\text{atm}}}$ is the normalized atmosphere spectral irradiance, and AW are the atmosphere transparency windows. It is shown that solar cell and Sun are electromagnetically active in a spectral range different from the one of radiative cooler and atmosphere.

We start our discussion with the hypothetical planar structure depicted in Figure 2A, based on the typical model used for the performance assessment of radiative coolers made of meta-materials (Perrakis et al., 2020, 2021; Safi and Munday, 2015; Zhu et al., 2014; Cagnoni et al., 2022). The device consists of a stack made by a reflector, a cement-based radiative cooler and a bifacial (Guerrero-Lemus et al., 2016) solar cell; the solar cell top surface is facing the Sun. This structure could be realized, for example, by building a thin film solar cell onto a cement-based substrate by sputtering, evaporation or solution deposition techniques. Experiments can be found in the literature, where thin-film solar cells have been placed onto structural elements of buildings such as roof tiles and concrete blocks (Águas et al., 2011; Iencinella et al., 2009; Hosseini et al., 2013).

By design, the solar cell and the radiative cooler are thermally coupled but mutually transparent. Indeed, the former absorbs sunlight in the UV-visible spectral range, whereas the latter emits thermal radiation in the IR spectral range, where the AW is found. Because absorbance and emissivity spectra of a body are equal according to Kirchhoff’s law of thermal radiation (Balaji, 2014) (they will be used interchangeably from this point onward), the solar cell and the radiative cooler do not exchange energy with each other electromagnetically, but do so only with the Sun and the atmosphere, separately. These observations are clarified in Figure 2B, where the most significant spectra are depicted (see Figure S1 for atmosphere transmittance spectra).

According to the considerations above, the device can be modeled as a single body at temperature *T*, whose electromagnetic properties are the ones of the solar cell in the UV-visible range and the ones of the radiative cooler in the IR spectral range (Safi and Munday, 2015). Then, one can calculate the net power density (power per top surface unit area) $P_{\text{net}}\left(T\right)$ exiting the device as function of *T* and determine the operating temperature by solving $P_{\text{net}}\left(T\right)=0$, which corresponds to the stationary state of the system. $P_{\text{net}}$ consists of several terms, as depicted in Figure 2A:

(Equation 1) $$P_{\text{net}}\left(T\right)=\underset{\text{UV}-\text{visible}\;\text{spectrum}}{\underset{︸}{P_{\text{rad}}^{\text{cell}}\left(T\text{,}E_{\text{g}}\right)-P_{\text{Sun}}\left(E_{\text{e},\lambda }^{\text{Sun}}\text{,}E_{\text{g}}\right)}}+P_{\text{elec}}\left(T\text{,}E_{\text{g}}\right)+\underset{\text{IR}\;\text{spectrum}}{\underset{︸}{P_{\text{rad}}^{\text{cool}}\left(T\text{,}A_{\text{Ω},\lambda }^{\text{cool}}\right)-P_{\text{atm}}\left(T_{0}\text{,}A_{\text{Ω},\lambda }^{\text{atm}}\text{,}A_{\text{Ω},\lambda }^{\text{cool}}\right)}}+P_{\text{con}}\left(T\text{,}T_{0}\text{,}h_{c}\right)$$

$P_{\text{Sun}}$, $P_{\text{rad}}^{\text{cell}}$ and $P_{\text{elec}}$ are the power densities that the solar cell absorbs from the Sun, emits as radiation and delivers to the end-user load at maximum power point (MPP), respectively; they have been calculated according to the Shockley-Queisser model (Shockley and Queisser, 1961). On the other hand, $P_{\text{atm}}$ and $P_{\text{rad}}^{\text{cool}}$ are the power densities that the radiative cooler absorbs from the atmosphere and emits as thermal radiation, respectively. Finally, $P_{\text{con}}$ is an empirical term to account for conduction and convection phenomena between the device and the environment. All these terms are widely discussed in the literature (Perrakis et al., 2020,^2021^; Safi and Munday, 2015; Zhu et al., 2014). Therefore, we refer the reader to the STAR methods section for their explicit formulas and we show here only their parametric dependencies, to highlight which information must be known to obtain $P_{\text{net}}$ as a function of *T* only.

In particular, some electromagnetic spectra must be supplied as a function of wavelength *λ* and zenith angle *θ* (the system is invariant with respect to the azimuth angle). The first one is the Sun spectral irradiance $E_{\text{e},\lambda }^{\text{Sun}}\left(\lambda \right)$, modeled using the global standard spectrum AM1.5g (ASTM International, 2022). The next one is the atmosphere spectral directional emissivity $A_{\text{Ω},\lambda }^{\text{atm}}\left(\lambda \text{,}\theta \right)$, obtained according to the formula $A_{\text{Ω},\lambda }^{\text{atm}}\left(\lambda \text{,}\theta \right)=1-T_{0,\lambda }^{\text{atm}}{\left(\lambda \right)}^{1/\text{cos}\left(\theta \right)}$(Perrakis et al., 2020), $T_{0,\lambda }^{\text{atm}}\left(\lambda \right)$ being the zero-zenith spectral directional transmittance calculated for the summer season with the software LOWTRAN (Hirsch, 2016). The last one is the spectral directional absorbance of the cement-based radiative cooler $A_{\text{Ω},\lambda }^{\text{cool}}\left(\lambda \text{,}\theta \right)$, calculated according to the workflow outlined in the Introduction and depicted in Figure 1.

The results originating from this workflow are the core subject of this section. However, before moving to their discussion, a few parameters of Equation 1 still need explanation. In particular, $E_{\text{g}}$ is the band-gap of the solar cell semiconductor, for which we have considered values in the range between 1 and 3eV, whereas $T_{0}$ and $h_{\text{c}}$ are the ambient temperature (set to 293.15K) and the conduction/convection coefficient (set to $10.6\;\text{W}\;{\text{m}}^{-2}\;{\text{K}}^{-1}$ to represent average winds (Perrakis et al., 2020)); different values of these two do not affect our findings, hence they are not considered here.

Back to $A_{\text{Ω},\lambda }^{\text{cool}}$, this can be readily determined by the transfer-matrix method (TMM) for a planar structure made of layers with known homogeneous permittivity (Born and Wolf, 2019). However, as already anticipated, common cement pastes are made of a heterogeneous mixture of chemical species arranged into a complex multi-scale porous structure (Dolado and Van Breugel, 2011). This apparent incompatibility can be lifted by resorting to a suitable effective medium theory (Choy, 2016), which enables us to substitute the microscopically inhomogeneous permittivity with a homogeneous one that provides equivalent electromagnetic properties at the macro-scale. This procedure requires knowledge of the cement paste micro-structure and of the complex permittivity of its homogeneous components, as well as a proper choice of the homogenization (effective medium) model. We are now going to discuss these aspects one by one.

### Micro-structure of the cement paste

Common cement pastes are prepared by mixing a fine powder (clinker) with water. This mixture undergoes a hydration process whose products form the cement paste, which gradually hardens over time. For OPCs, the powder is made of alite, whose chemical formula is ${\text{Ca}}_{3}{\text{SiO}}_{5}$ (${\text{C}}_{3}\text{S}$ in cement chemist notation), by up to 70% (Taylor, 2004). Accordingly, we have considered a cement paste made by hydrating alite only, to simplify the model while capturing all the essential features of OPCs.

**Figure 3:**
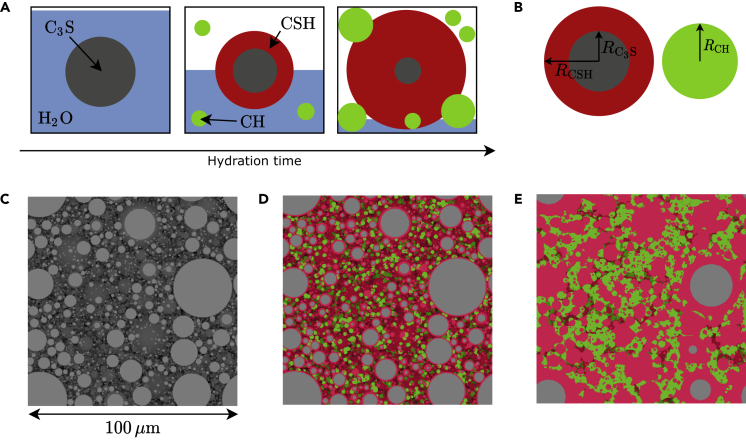
Simulation of the alite hydration process for an initial particle size distribution corresponding to $N_{0}=10^{5}$ particles per ${\left(100\;\mu \text{m}\right)}^{3}$ (A) Sketch of the hydration process of a single alite particle representing the model used in this work. (B) Definition of the radii used to quantify the particles size in this work. (C) Simulation snapshot of the initial cement powder. (D) Simulation snapshot of the partially hydrated cement paste. (E) Simulation snapshot of the fully hydrated cement paste.

The surface of the alite powder particles, which are typically assumed to be spherical (Navi and Pignat, 1996), dissolves on reaction with water. The dissolution products form shells of calcium silicate hydrate, whose chemical formula is ${\left(\text{CaO}\right)}_{3}{\left({\text{SiO}}_{2}\right)}_{2}{\left({\text{H}}_{2}\text{O}\right)}_{4}$ (CSH in cement chemist notation), around the original particles, or form new particles made of portlandite, whose chemical formula is $\text{Ca}{\left(\text{OH}\right)}_{2}$ (CH in cement chemist notation), by nucleation and growth in the interstitial regions. This process is sketched in Figure 3A and follows the volumetric formula (Pignat et al., 2005):

(Equation 2) $$1.0\;V_{{\text{C}}_{3}\text{S}}+1.318\;V_{{\text{H}}_{2}\text{O}}\to 1.57\;V_{\text{CSH}}+0.596\;V_{\text{CH}}$$

The resulting cement paste is made of a disordered ensemble of two kinds of domains, namely the ones with a core of ${\text{C}}_{3}\text{S}$ and a shell of CSH, and the ones made of CH only, characterized by a pseudo-spherical shape.

To generate plausible cement micro-structures, we have simulated the alite hydration process with the open source package *μ*ic (Bishnoi and Scrivener, 2009a). In particular, we have applied the well-established model from Pignat et al. (2005) to a specimen filled with a 0.4 water/${\text{C}}_{3}\text{S}$ mass ratio, in line with common cement recipes, and considered initial particle size distributions (PSDs) for the alite powder corresponding to $N_{0}=10^{2},10^{3},10^{4},10^{5},10^{6},10^{7}$ particles per ${\left(100\;\mu \text{m}\right)}^{3}$, to unravel the interplay between properties and micro-structure. The implementation of this model in *μ*ic is well described in the doctoral thesis by Bishnoi (2008); for the reader’s convenience, we have reported a detailed description in the STAR methods section.

Table 1 reports information concerning the final chemical composition, characterized by the volume fractions *f*, and the resulting particle size statistics, quantified by the expectation value *μ* and the standard deviation *σ* of the radii of the ${\text{C}}_{3}\text{S}$ cores, the CSH shells, and the CH particles; the definition of the radii is shown in Figure 3B. Figure 3 also shows three simulation snapshots for $N_{0}=10^{5}$ particles per ${\left(100\;\mu \text{m}\right)}^{3}$, corresponding to the initial cement powder (C), the partially hydrated cement paste (D) and the fully hydrated cement paste (E). As expected, a larger value of $N_{0}$ leads to smaller final sub-domains. More importantly, the size of these sub-domains is comparable to the thermal radiation wavelengths. Therefore, micro-structural size effects are expected to impact the emissivity properties of the samples, hence providing a knob to tune the dielectric response. Finally, it can be seen from the values of $f_{{\text{C}}_{3}\text{S}}$ (≤5%) that alite is consumed almost completely on full hydration and can be neglected in the homogenization step.

**Table 1:** Initial number of particles per ${\left(100\;\mu \text{m}\right)}^{3}$$N_{0}$, volume fractions *f* and statistics (expectation values *μ* and standard deviations *σ*) of the particle radii *R* of the simulated samples after full hydration

$\frac{N_{0}}{{\left(100\;\mu \text{m}\right)}^{-3}}$	$\frac{f_{{\text{C}}_{3}\text{S}}}{\text{\%}}$	$\frac{f_{\text{CSH}}}{\text{\%}}$	$\frac{f_{\text{CH}}}{\text{\%}}$	$\frac{\mu \left[R_{{\text{C}}_{3}\text{S}}\right]}{\mu \text{m}}$	$\frac{\mu \left[R_{\text{CSH}}\right]}{\mu \text{m}}$	$\frac{\mu \left[R_{\text{CH}}\right]}{\mu \text{m}}$	$\frac{\sigma \left[R_{\text{C}3\text{S}}\right]}{\mu \text{m}}$	$\frac{\sigma \left[R_{\text{CSH}}\right]}{\mu \text{m}}$	$\frac{\sigma \left[R_{\text{CH}}\right]}{\mu \text{m}}$
$10^{2}$	5	61	23	3.32	12.51	21.94	2.80	1.82	2.49
$10^{3}$	5	62	24	0.71	5.20	11.33	1.34	1.89	2.00
$10^{4}$	4	63	24	0.11	1.85	5.04	0.42	1.12	1.18
$10^{5}$	4	63	24	0.01	0.58	2.27	0.12	0.52	0.58
$10^{6}$	4	64	24	0.00	0.17	0.86	0.03	0.21	0.56
$10^{7}$	2	66	25	0.00	0.10	0.36	0.01	0.09	0.28

The sum of the volume fractions is not unity because the remaining space consists of empty pores. The definition of the radii is depicted in Figure 3B.

### Complex permittivity of the cement paste components

To apply effective medium theory, the micro-structural information obtained above must be combined with the complex permittivity of the cement paste homogeneous components, namely ${\text{C}}_{3}\text{S}$, CSH and CH. Because atomic vibrations are responsible for the dielectric response in the IR spectral range where the radiative cooler operates, we have resorted to molecular simulations to obtain this information.

We have performed these simulations with the General Utility Lattice Program (GULP) (Gale, 1997) according to the force field method (Leach, 2001). Under this atomistic scheme, the interaction between atoms is described by parameterized interatomic potentials so that the system energy can be traced back from their positions. In particular, we have adopted a well-tested polarizable force field, which is known to describe correctly the structure and elastic properties of most cementitious phases (Manzano et al., 2009). Later, we have calculated the complex permittivity of ${\text{C}}_{3}\text{S}$, CSH and CH by following the method employed in (Dolado et al., 2020) for studying the response of cement-based materials in the THz regime.

**Figure 4:**
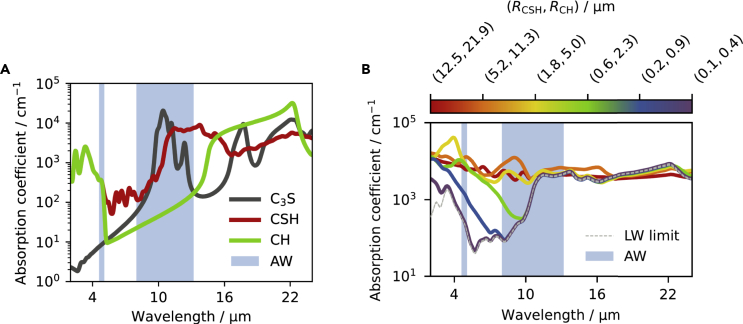
Absorption spectra of the materials studied (A) Calculated absorption coefficient of the homogeneous components of cement pastes made by hydrating alite powder. (B) Effective absorption coefficient of the generated cement pastes as a function of the mean particle (sub-domain) radii $\left(R_{\text{CSH}}\text{,}R_{\text{CH}}\right)$.

The corresponding absorption coefficient is reported in Figure 4A for all components. Details about molecular simulations and crystal structures can be found in the STAR methods section, whereas Figure S2 - drawn with VESTA (Momma and Izumi, 2011) - and Table S1 report information on the unit cells. Remarkably, the broad absorption maximum of CSH, which is the component with the highest volume fraction (see Table 1), overlaps significantly with the AW, as desired. At the same time, all the components exhibit significant absorption outside the AW. This is an advantage for solar cell applications, where the supra-ambient device operating temperature ensures that the radiative cooler is going to eject more energy than the one received from the atmosphere also outside of the AW, hence enhancing the cooling performance (Zhao et al., 2019). Finally, although the absorption coefficient ($∼10^{4}{\text{cm}}^{-1}$) is not as strong as the one of typical thin-film absorbers ($∼10^{6}{\text{cm}}^{-1}$), the possible realization of thick geometries still allows us to achieve high absorbance, hence removing this apparent weakness.

### Complex permittivity of the cement paste

The ingredients required to estimate the complex permittivity of the generated cement pastes by a suitable effective medium theory are now available. Care should be taken because Bruggeman’s formula (Bruggeman, 1935), which is the most commonly used model for heterogeneous materials, does not account for micro-structure size effects. Indeed, it is derived in the long-wavelength (LW) limit, *i.e.*, under the assumption that the particles size is much smaller than the wavelengths of interest. However, we have seen above that the size of the cement sub-domains is comparable to the wavelengths around the AW. Therefore, a model capable of capturing the main effects of micro-structure on the dielectric properties is needed to properly estimate the effective complex permittivity of our cement pastes. Accordingly, we have used a recent model proposed by Slovick (2017) for a disordered ensemble of spherical particles with a space filling matrix of infinitesimally small spheres. This is representative of our cement paste, made of a high-density disordered network of CSH and CH spheres with a mean radius dependent on the alite initial PSD according to Table 1, separated by empty interstices (pores). According to Slovick’s model, the effective complex permittivity $\varepsilon_{\text{eff}}$ of this system can be obtained by solving the equation:

(Equation 3) $$f_{\text{CSH}}\;\frac{\varepsilon_{\text{CSH}}\;F\left(k_{\text{CSH}}\;R_{\text{CSH}}\right)-\varepsilon_{\text{eff}}}{\varepsilon_{\text{CSH}}\;F\left(k_{\text{CSH}}\;R_{\text{CSH}}\right)+2\;\varepsilon_{\text{eff}}}+f_{\text{CH}}\;\frac{\varepsilon_{\text{CH}}\;F\left(k_{\text{CH}}\;R_{\text{CH}}\right)-\varepsilon_{\text{eff}}}{\varepsilon_{\text{CH}}\;F\left(k_{\text{CH}}\;R_{\text{CH}}\right)+2\;\varepsilon_{\text{eff}}}+f_{\text{air}}\;\frac{1-\varepsilon_{\text{eff}}}{1+2\;\varepsilon_{\text{eff}}}=0$$

where *f*, *ε* and *k* are volume fraction, complex permittivity and complex wave vector, and $F\left(x\right)=2\;\left(\text{sin}x-x\;\text{cos}x\right)/\left[x\;\text{cos}x+\left(x^{2}-1\right)\;\text{sin}x\right]$ (Belyaev and Tyurnev, 2018). It is worth noting that Equation 3 becomes Bruggeman’s formula in the LW limit becauseF(x→0)→1. Furthermore, by making no distinction between background medium and inclusions, the formula is valid for any volume fraction, contrarily to Maxwell-Garnett formula, which is valid only for a low volume fraction of the inclusions (Markel, 2016).

Figure 4B reports the obtained cement paste absorption coefficient as a function of the mean particle (sub-domain) radii $\left(R_{\text{CSH}}\text{,}R_{\text{CH}}\right)$. The spectra exhibit increased absorption at wavelengths comparable to the particles size, because of the multiple reflections occurring at the particle boundaries, leading to interference effects and enhanced absorption lengths, similarly to the case of planar layers. As the particles become smaller, the additional absorption band shifts toward the UV-visible range (shorter wavelength) and, eventually, size effects become negligible in the IR range, which can then be described in the LW limit (see the case $\left(R_{\text{CSH}}\text{,}R_{\text{CH}}\right)=\left(0.1\text{,}0.4\right)\;\mu \text{m}$). The eventual additional emissivity in the UV-visible range is not going to impact the radiative cooler performance, because its radiated spectral power density is given by the product of its emissivity spectrum (≤1) with the spectral power density radiated by a black-body (Balaji, 2014), which is negligible in the UV-visible spectrum at terrestrial temperatures.

These results show that the absorption properties of the cement paste can be tuned by modifying its micro-structure and tailored to the spectral requirements of radiative cooling. As a matter of fact, a similar approach has been applied to hierarchical porous polymers mentioned in the Introduction (Mandal et al., 2018).

Although this is very promising, care should be taken. Indeed, modeling the cement paste micro-structure as an ensemble of air-embedded spherical inclusions is a geometrical approximation because these are eventually going to “collide” during growth and partially “deform”. In addition, effective medium theories assume that all particles are subject to the same mean field. Deviations might occur close to the percolation threshold. These aspects should be investigated in future studies.

These results are extremely encouraging, but not enough. Indeed, a larger absorption coefficient does not necessarily imply a stronger absorbance because it stems from a larger extinction coefficient that might also increase the layer reflectance at the same time. Therefore, more radiation could be lost by reflection before even entering the cement paste, hence leading to a reduced absorbance, as we shall see in a moment.

### Emissivity of the cement paste

We have used the effective complex permittivity $\varepsilon_{\text{eff}}$ calculated above to determine the spectral directional absorbance $A_{\text{Ω},\lambda }^{\text{cool}}\left(\lambda \text{,}\theta \right)$ of layers made of the generated cements by the transfer-matrix method. We have considered cement slabs with a thickness of 100μm. Indeed, based on the absorption coefficient values reported in Figure 4B, nanometer-scale thicknesses, although experimentally feasible (Rheinheimer and Casanova, 2012), are unsuitable to obtain large absorbance/emissivity because of the lack of absorption strength. This is clearly not an issue with cement-based solutions, for which very thick geometries are usually preferred. As a matter of fact, we are opting for a rather thin cement layer, which corresponds to a worst case scenario and makes our assessment stronger. Furthermore, this choice let us draw conclusions also about concrete, where aggregates are going to break the cement paste continuity and make the effective thickness smaller. Finally, this thickness has been experimentally achieved in the literature (Zhang et al., 2016).

Implementation-wise, we have adopted a generalized TMM capable of describing incoherent propagation through layers having rough surfaces with Gaussian disorder (Katsidis and Siapkas, 2002; Centurioni, 2005). This model is representative of cement slabs, typically characterized by randomly disordered surfaces with roughness values from a few hundred nanometers to a few micrometers, depending on polishing (Apedo et al., 2015). This kind of rough surfaces can provide an advantage in terms of emissivity, because they have been shown to reduce reflection and increase absorption of an incoming electromagnetic wave by randomizing its direction and enlarging the effective absorption length (light-trapping) (Yablonovitch, 1982; Kowalczewski et al., 2012; Cappelluti et al., 2018).

Our calculations are in agreement with these statements and show that a rougher surface slightly increases the absorbance of our cement slabs. Because of their supplementary nature, these results on the effect of surface roughness are shown in Figure S3 and only the worst case of a flat surface is considered in the main text. The STAR methods section contains a detailed description of the generalized TMM used.

**Figure 5:**
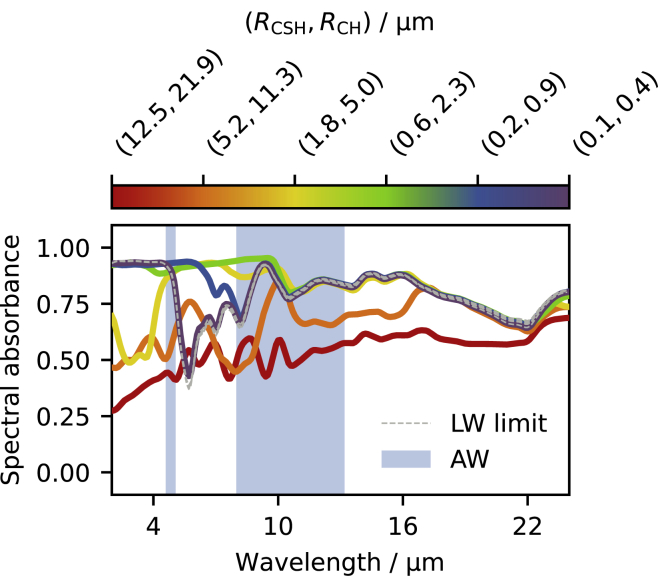
Spectral absorbance (angular-average) of the cement layer as a function of the mean particle (sub-domain) radii $\left(R_{\text{CSH}}\text{,}R_{\text{CH}}\right)$

Figure 5 shows the angular-average of the spectral absorbance (emissivity) of the cement layer as a function of the mean particle (sub-domain) radii $\left(R_{\text{CSH}}\text{,}R_{\text{CH}}\right)$. As anticipated, the samples absorbance and absorption coefficient (Figure 4B) are not trivially related. For example, the sample with the largest sub-domains exhibit a significantly smaller absorbance, which cannot be readily inferred from the absorption coefficient but is because of an increased reflectance (see Figure S4). Interestingly, these results show that it is possible to broaden the emission spectrum of the cement paste and approach the desired black-body-like IR emissivity by decreasing the sub-domains size. However, if the particles become too small, the spectral absorbance departs again from target. Optimal CSH radii seem to be in the 0.5−2.0μm range. Remarkably, these results are in good agreement with recent experiments (Lu et al., 2021).

### Radiative cooling performance

Finally, we have evaluated the cement paste radiative cooling performance by solving Equation 1 with respect to *T*. In particular, we have determined the operating temperature of the device depicted in Figure 2A as a function of the band-gap of the solar cell semiconductor. We have also studied the scenario where no radiative cooler is used, which provides a benchmark against which to compare to quantify the effectiveness of the cement-based radiative cooler.

**Figure 6:**
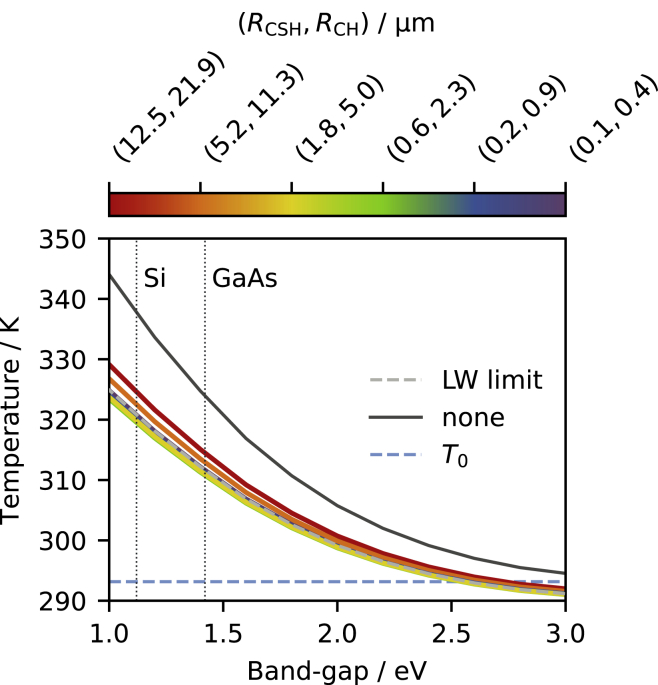
Solar cell temperature reduction by cement-based radiative cooling The plot shows the operating temperature versus the solar cell semiconductor band-gap for the device depicted in Figure 2A as a function of the mean particle (sub-domain) radii $\left(R_{\text{CSH}}\text{,}R_{\text{CH}}\right)$, calculated by solving Equation 1. The cooler-free case is also reported for comparison. The band-gaps corresponding to crystalline silicon (Si) and gallium arsenide (GaAs), on which today’s best performing solar cells are based, are highlighted.

The obtained temperature vs band-gap curves are reported in Figure 6. Remarkably, our results suggest that a radiative cooler made of a cement paste produced by alite hydration could significantly reduce the operating temperature of a solar cell. For example, the temperature of Si-based solar cells is reduced by approximately 20K. According to the thumb rules reported in the introduction, this could correspond to up to 9% efficiency gain and 4-fold lifetime extension. In agreement with the absorbance spectra shown in Figure 5, reducing the size of the cement sub-domains leads to superior cooling performance, as long as the particles size does not become too small and the LW limit is approached. As stated in the previous section, optimal CSH radii seem to be in the 0.5−2.0μm range.

### Conclusions and outlook

In summary, we have computed the thermal emissivity properties of cement pastes made by alite hydration as a function of their micro-structure. Our results demonstrate that slabs made of these materials exhibit strong thermal emission in the IR spectrum around the AW, in agreement with recent experimental work (Lu et al., 2021). The emissivity spectrum approaches the one of an IR black-body if the size of the sub-domains making up the micro-structure is properly engineered. This corresponds to the ideal IR spectral target for radiative coolers applied to solar cells. Accordingly, we have also studied the thermodynamic efficiency limit of solar cells coupled with cement-based radiative coolers for the first time and found that this solution exhibits outstanding potential, with a possible reduction of the operating temperature of silicon-based solar cells by up to 20K. This value could provide impressive performance gains in power conversion efficiency (up to 9%) and lifetime (up to 400%).

Considered the reliability and stability of these materials and the incredibly low cost of the raw elements needed to produce them, cements and (meta-)concretes appear as ideal candidates to fulfill all the performance, scalability and reliability requirements that would turn radiative cooling into an energetically efficient, economical viable, environmentally friendly, and reliable technology for the thermal management of solar cells.

Therefore, this new line of research deserves further exploration aimed at addressing the main open challenges for its practical implementation, which we briefly outline as a possible road-map. First, the electromagnetic properties could be improved even further by working on the cement chemistry and micro-structure, or by inserting suitable aggregates into the cement paste. Second, the heat transfer from the solar cell to the cement slab needs to be fully characterized and optimized to ensure efficient thermal flow between the two. Finally, a practical device should be designed based on multi-physics simulations and experimentally fabricated and characterized to ultimately prove the effectiveness of this solution.

At this stage, we have no reason to believe that some of these aspects cannot be tackled by science and technology. Indeed, both the chemical and the micro-structural landscapes of cements and concretes can be tuned to a large extent by modifying the precursor materials or the preparation protocols, leading to very diverse properties (Aïtcin, 2000; Bensted and Barnes, 2002; Bohnet and Ullmann, 2003). Furthermore, many techniques and configurations exist for the placement of cements and concretes (structural and non-structural), from conventional building construction methods (Gagg, 2014), 3D printing (Zhang et al., 2019) and spraying (Austin and Robins, 2010), to machine-based thinning (down to 100μm) (Zhang et al., 2016) and nanometer thin-film deposition (Rheinheimer and Casanova, 2012). This flexibility enables one to envision many options for the realization of the structure depicted in Figure 2A. As a matter of fact, thin-film solar cells have already been placed onto building structural elements (roof tiles, concrete blocks) in previous experimental studies (Águas et al., 2011; Iencinella et al., 2009; Hosseini et al., 2013), although with no regard for radiative cooling, *i.e.*, without optimization of the materials and system properties of interest, but in the context of building-integrated photovoltaics. These works can provide a starting point for the practical implementation of our attractive concept and pave the way toward more efficient photovoltaic systems.

### Limitations of the study

Although the calculated material properties are consistent with experimental data from the literature, the experimental realization of a solar cell with a cementitious radiative cooler is needed to confirm our computational predictions.

## STAR★Methods

### Key resources table

**Table:** 

REAGENT or RESOURCE	SOURCE	IDENTIFIER
**Software and algorithms**		

LOWTRAN	(Hirsch, 2016)	https://zenodo.org/record/213475
μic	(Bishnoi and Scrivener, 2009a)	https://micepfl.sourceforge.net/index.html
General Utility Lattice Program (GULP)	(Gale, 1997)	http://gulp.curtin.edu.au/gulp/
VESTA	(Momma and Izumi, 2011)	http://www.jp-minerals.org/vesta/en/

### Resource availability

#### Lead contact

Further information and requests for resources should be directed to and will be fulfilled by Matteo Cagnoni (matteo.cagnoni@polito.it).

#### Materials availability

This study did not generate new unique reagents.

### Method details

#### Device power balance model

We have calculated the terms of Equation 1 by adopting a spherical coordinate system whose origin is on the top surface of the device in Figure 2A. The zenith angle *θ* is measured with respect to the direction normal to this surface, while the system properties are invariant with respect to the azimuth angle. The expressions are:

$$P_{\text{Sun}}\left(E_{\text{e},\lambda }^{\text{Sun}}\text{,}E_{\text{g}}\right)=\int_{0}^{h\;c/E_{\text{g}}}\text{d}\lambda \;E_{\text{e},\lambda }^{\text{Sun}}\left(\lambda \right)$$

$$P_{\text{rad}}^{\text{cell}}\left(T\text{,}E_{\text{g}}\right)=\pi \;\int_{0}^{h\;c/E_{\text{g}}}\text{d}\lambda \;L_{\text{e},\text{Ω},\lambda }^{\text{BB}}\left(\lambda \text{,}T\text{,}V_{\text{MPP}}\left(T\text{,}E_{\text{g}}\right)\right)$$

$$P_{\text{elec}}\left(T\text{,}E_{\text{g}}\right)=J_{\text{MPP}}\left(T\text{,}E_{\text{g}}\right)\;V_{\text{MPP}}\left(T\text{,}E_{\text{g}}\right)$$

$$P_{\text{atm}}\left(T_{0}\text{,}A_{\text{Ω},\lambda }^{\text{atm}}\text{,}A_{\text{Ω},\lambda }^{\text{cool}}\right)=\int_{-\pi }^{+\pi }\text{dΩ}\;\int_{0}^{+\infty }\text{d}\lambda \;\text{cos}\theta \;A_{\text{Ω},\lambda }^{\text{atm}}\left(\lambda \text{,}\theta \right)\;A_{\text{Ω},\lambda }^{\text{cool}}\left(\lambda \text{,}\theta \right)\;L_{e,\text{Ω},\lambda }^{\text{BB}}\left(\lambda \text{,}T_{0}\text{,}0\right)$$

$$P_{\text{rad}}^{\text{cool}}\left(T\text{,}A_{\text{Ω},\lambda }^{\text{cool}}\right)=\int_{-\pi }^{+\pi }\text{dΩ}\;\int_{0}^{+\infty }\text{d}\lambda \;\text{cos}\theta \;A_{\text{Ω},\lambda }^{\text{cool}}\left(\lambda \text{,}\theta \right)\;L_{e,\text{Ω},\lambda }^{\text{BB}}\left(\lambda \text{,}T\text{,}0\right)$$

$$P_{\text{con}}\left(T\text{,}T_{0}\text{,}h_{c}\right)=h_{c}\;\left(T-T_{0}\right)$$

where $L_{e,\text{Ω},\lambda }^{\text{BB}}$ is the spectral directional radiance of a black-body at temperature *T* with an applied voltage *V* (Würfel, 1982):

$$L_{e,\text{Ω},\lambda }^{\text{BB}}\left(\lambda \text{,}T\text{,}V\right)=\frac{2\;h\;c^{2}}{\lambda^{5}}\frac{1}{\text{exp}\left(\frac{h\;c/\lambda -q\;V}{k\;T}\right)-1}$$

$J_{\text{MPP}}$ and $V_{\text{MPP}}$ are the solar cell electric current density and output voltage at maximum power point (MPP), respectively, calculated with the Shockley-Queisser model. *λ*, Ω, *h*, *c*, *k* and *q* are wavelength, solid angle, Planck’s constant, speed of light in vacuum, Boltzmann’s constant and elementary charge, respectively. The other symbols have been defined in the main text. The reader is referred to the literature for more details concerning the definition of the radiometry quantities introduced above (Balaji, 2014).

#### Alite hydration model

We have simulated the alite hydration process depicted in Figure 3 by implementing the model from Pignat et al. (Pignat et al., 2005) into the open-source chemical kinetics package *μ*ic (Bishnoi and Scrivener, 2009a), as done by Bishnoi in his doctoral thesis (Bishnoi, 2008). We have considered a cubic specimen of 100μm side length with periodic boundary conditions initially filled with a continuum of water and discrete ${\text{C}}_{3}\text{S}$ spherical particles in a water/${\text{C}}_{3}\text{S}$ mass ratio of 0.4 (Bishnoi and Scrivener, 2009b), in line with common cement recipes. We have considered different initial particle size distributions (PSDs), which were provided with the software, corresponding to an initial number of alite particles $N_{0}$ within the 100μm-side-length cube equal to $10^{2}$, $10^{3}$, $10^{4}$, $10^{5}$, $10^{6}$ and $10^{7}$, so that we could investigate the role of micro-structure in the determination of the radiative cooling properties. The rate of the hydration process, which is described by the volumetric formula given in Equation 2 under a mass density of $3.15\;{\text{g cm}}^{-3}$ for ${\text{C}}_{3}\text{S}$, $2.0\;{\text{g cm}}^{-3}$ for CSH and $2.24\;{\text{g cm}}^{-3}$ for CH, is controlled by the decrease in size of the ${\text{C}}_{3}\text{S}$ particles. In turn, this is related to the formation rate of CSH and CH. CSH shells form onto the ${\text{C}}_{3}\text{S}$ particles surface by the combination of three mechanisms, namely a nucleation and growth mechanism, a phase boundary mechanism, and a diffusion controlled mechanism. The corresponding equations are:

$$\frac{\text{d}R_{{\text{C}}_{3}\text{S}}}{\text{d}t}=-3\;k_{1}\;t^{2}\;\text{exp}\left(-k_{1}\;t^{3}\right)$$

$$\frac{\text{d}R_{{\text{C}}_{3}\text{S}}}{\text{d}t}=-k_{2}$$

$$\frac{\text{d}R_{{\text{C}}_{3}\text{S}}}{\text{d}t}=-\frac{k_{3}}{R_{\text{CSH}}-R_{{\text{C}}_{3}\text{S}}}$$

where $k_{1}=1.14\times 10^{-4}\;{\text{h}}^{-3}$, $-k_{2}$ equals the minimum of the right-hand-side of the first equation, and $k_{3}=0.01\;\mu {\text{m}}^{2}\;{\text{h}}^{-1}$ (Pignat et al., 2005; Bishnoi, 2008). At the same time, new CH particles form at an exponentially decreasing nucleation rate in the interstitial regions of the hydrating cement paste according to the formula

$$n\left(t\right)=n_{\text{max}}\left(1-\text{exp}\left(-a\;t\right)\right)$$

where $n_{\text{max}}$ is set to one-fifth of $N_{0}$ (Navi and Pignat, 1996) and $a=0.213\;{\text{h}}^{-1}$ (Jennings and Parrott, 1986). Their growth occurs randomly but constrained by the amount of product available according to the hydration reaction rate.

#### Molecular simulations and crystal structures

To obtain the IR dielectric properties of the cement paste components, we have performed molecular simulations with the General Utility Lattice Program (GULP) (Gale, 1997) according to the force field method (Leach, 2001), by adopting a well-tested polarizable force field, which is known to describe correctly the structure and elastic properties of most cementitious phases (Manzano et al., 2009).

For the atomistic structure of the almost amorphous CSH, we have employed the model proposed in (Dolado et al., 2020) and (Duque Redondo, 2018), which corresponds to a very large system whose exact stoichiometry is ${\left(\text{CaO}\right)}_{254}{\left({\text{SiO}}_{2}\right)}_{152}{\left({\text{H}}_{2}\text{O}\right)}_{306}$. For the crystalline structures of ${\text{C}}_{3}\text{S}$ and CH, we have relaxed the experimental unit cells measured in (Mumme, 1995) and (Desgranges et al., 1993), respectively. The stoichiometry and the final simulation cell parameters are disclosed in Table S1. The unit cells drawn with the software VESTA (Momma and Izumi, 2011) are depicted in Figure S2.

Later, we have calculated the complex permittivity of ${\text{C}}_{3}\text{S}$, CSH and CH by following the method employed in (Dolado et al., 2020) for studying the response of cement-based materials in the THz regime. The method resorts to expressing the dielectric tensor in terms of the oscillator strengths of the vibrational modes as

$$\varepsilon_{ij}\left(\omega \right)=\varepsilon_{ij}\left(+\infty \right)+\frac{4\;\pi }{V}\sum_{m}\frac{{\text{Ω}}_{ij}^{m}}{\omega_{m}^{2}-\omega^{2}}$$

where *ω* is the angular frequency, *V* is the unit cell volume, *m* is the phonon mode rank, and $\omega_{m}$ are the mode-specific frequencies. The oscillator strength tensor for each vibrational mode *m* depends on the Born effective charges $q^{\text{B}}$ and the eigenvector $e_{ij}$ for that mode according to

$${\text{Ω}}_{\alpha \beta }=\left(\sum_{i}^{N}\frac{q_{i\alpha j}^{\text{B}}\;e_{ij}}{\sqrt{m_{i}}}\right)\left(\sum_{i}^{N}\frac{q_{i\beta j}^{\text{B}}\;e_{ij}}{\sqrt{m_{i}}}\right)$$

with $m_{i}$ denoting the ion masses. In the practical computational implementation, we have used a small damping term *δ* of $10\;{\text{cm}}^{-1}$ by the substitution $\omega^{2}\to \omega \left(\omega +i\;\delta \right)$. Finally, we have averaged the principal components of the dielectric tensor, *i.e.*, we have taken $\varepsilon \left(\omega \right)=\sum_{i=1}^{3}\varepsilon_{i}\left(\omega \right)/3$, in agreement with the disordered micro-structural nature of cements, which lifts off any preferred orientation.

#### Generalized transfer-matrix-method

We have used a generalized form of the transfer-matrix method capable of describing incoherent propagation and rough surfaces. In particular, the surface height variation is supposed to follow a Gaussian probability distribution with a given root-mean-square value (RMS) (Katsidis and Siapkas, 2002; Centurioni, 2005).

The amplitude *E* of the electric fields propagating from left to right (+) and right to left (−) on the left (L) and right (R) sides of the layer are related by the formula:

$$\left(\begin{array}{l} {\left|E_{\text{L}}^{+}\right|}^{2}\\ {\left|E_{\text{L}}^{-}\right|}^{2} \end{array}\right)=\left(D_{\text{L}}\;P\;D_{\text{R}}\right)\;\left(\begin{array}{l} {\left|E_{\text{R}}^{+}\right|}^{2}\\ {\left|E_{\text{R}}^{-}\right|}^{2} \end{array}\right)$$

where $D_{\text{L}}$ and $D_{\text{R}}$ describe the field propagation across the left and right interfaces between the layer and vacuum, respectively, and **P** across the layer. Their formulas are

$$D=\frac{1}{{\left|t_{\text{lr}}\right|}^{2}}\;\left(\begin{array}{ll} 1 & -{\left|r_{\text{rl}}\right|}^{2}\\ {\left|r_{\text{lr}}\right|}^{2} & {\left|t_{\text{lr}}\;t_{\text{rl}}\right|}^{2}-{\left|r_{\text{lr}}\;r_{\text{rl}}\right|}^{2} \end{array}\right)$$

$$P=\left(\begin{array}{ll} {\left|\text{exp}\;\left(-i\;\delta \right)\right|}^{2} & 0\\ 0 & {\left|\text{exp}\;\left(i\;\delta \right)\right|}^{2} \end{array}\right)$$

with

$$r_{\text{lr}}=r_{\text{lr}}^{\left(0\right)}\;\text{exp}\left[-2{\left(\frac{2\;\pi \;\text{RMS}\;N_{\text{l}}}{\lambda }\right)}^{2}\right]$$

$$t_{\text{lr}}=t_{\text{lr}}^{\left(0\right)}\;\text{exp}\left[-\frac{1}{2}{\left(\frac{2\;\pi \;\text{RMS}}{\lambda }\right)}^{2}{\left(N_{\text{r}}-N_{\text{l}}\right)}^{2}\right]$$

$$\delta =2\;\pi \;\frac{N\;d\;\text{cos}\theta }{\lambda }$$

The subscripts l and r denote left and right sides of the interface, while *lr* means from the left to the right side of the interface. A graphical representation of the quantities introduced can be found in Figure S5.

$r_{\text{lr}}^{\left(0\right)}$ and $t_{\text{lr}}^{\left(0\right)}$ are the Fresnel reflection and transmission coefficients of a smooth planar surface (for which we have used the average between the *s* and *p* polarization values), *N* is the complex refractive index, *d* is the layer thickness, and *θ* is the refraction angle.

The spectral directional reflectance, transmittance and absorbance are readily obtained, respectively, from the following formulas:

$$R_{\text{Ω},\lambda }\left(\lambda ,\theta \right)=\frac{{\left(D_{\text{L}}\;P\;D_{\text{R}}\right)}_{21}}{{\left(D_{\text{L}}\;P\;D_{\text{R}}\right)}_{11}}$$

$$T_{\text{Ω},\lambda }\left(\lambda ,\theta \right)=\frac{1}{{\left(D_{\text{L}}\;P\;D_{\text{R}}\right)}_{11}}$$

$$A_{\text{Ω},\lambda }\left(\lambda \text{,}\theta \right)=1-R_{\text{Ω},\lambda }\left(\lambda \text{,}\theta \right)-T_{\text{Ω},\lambda }\left(\lambda \text{,}\theta \right)$$

The (hemispherical) spectral reflectance, transmittance and absorbance are simply the angular average of their spectral directional counterparts. For example, the spectral reflectance is calculated as follow for a system with azimuthal invariance such as the device depicted in Figure 2A:

$$R_{\lambda }\left(\lambda \right)=2\int_{0}^{\pi /2}\text{d}\theta \;R_{\text{Ω},\lambda }\left(\lambda \text{,}\theta \right)\;\text{cos}\left(\theta \right)\;\text{sin}\left(\theta \right)$$

## Data Availability

• All data reported in this paper will be shared by the lead contact upon request. • This paper does not report original code. • Any additional information required to reanalyze the data reported in this paper is available from the lead contact upon request.
