# Supplementary material for: Cementitious materials as promising radiative coolers for solar cells
Source: iScience. 2022 Oct 13;25(11):105320. doi: 10.1016/j.isci.2022.105320 (PMC9615327; doi:10.1016/j.isci.2022.105320)
Supplement: Document S1. Figures S1–S5 and Table S1 [file mmc1.pdf]

iScience, Volume *25*

Supplemental information

Cementitious materials as promising radiative coolers for solar cells

Matteo Cagnoni, Alberto Tibaldi, Jorge S. Dolado, and Federica Cappelluti

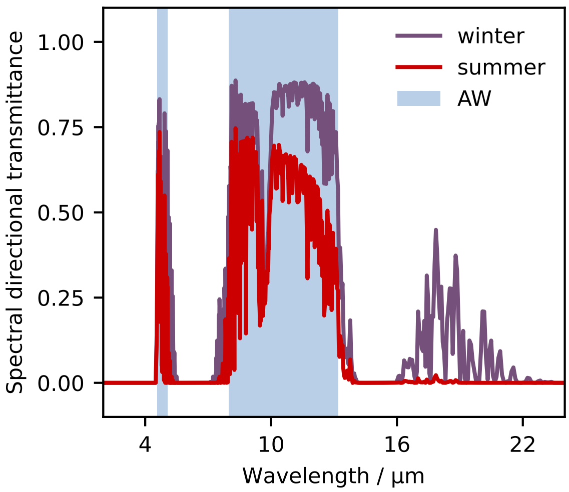

Figure S1: Comparison between the atmosphere spectral directional transmittance at zero-zenith during summer and winter seasons, and atmospheric windows. Related to **Figure 2b**.

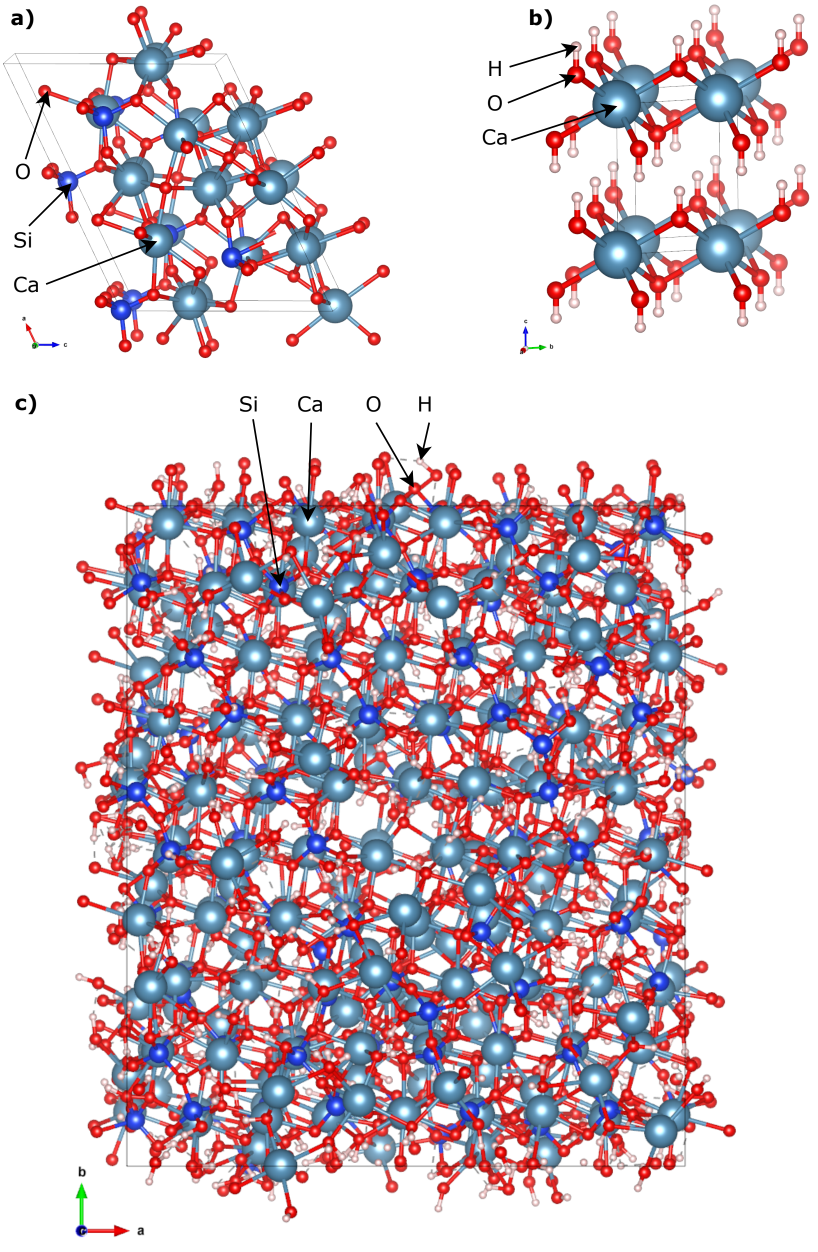

Figure S2: Unit cells used in the molecular simulations, displayed with the software VESTA. Related to **STAR Methods**.
(A) C_3S.
(B) CH.
(C) CSH.

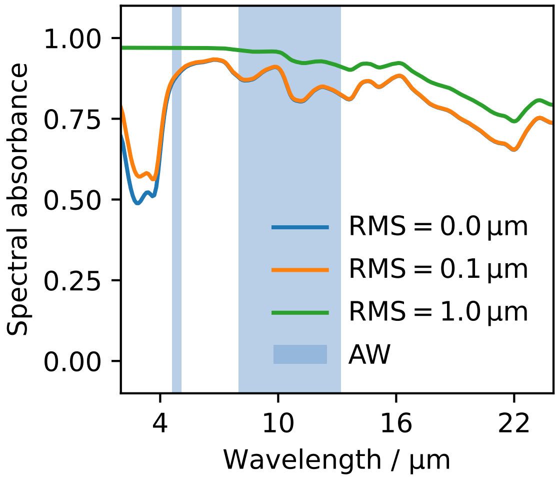

Figure S3: Effect of surface roughness on the cement layer spectral absorbance for $N_0 = 10^4$ $(100\mu\mathrm{m})^{-3}$. Related to **Figure 5**.

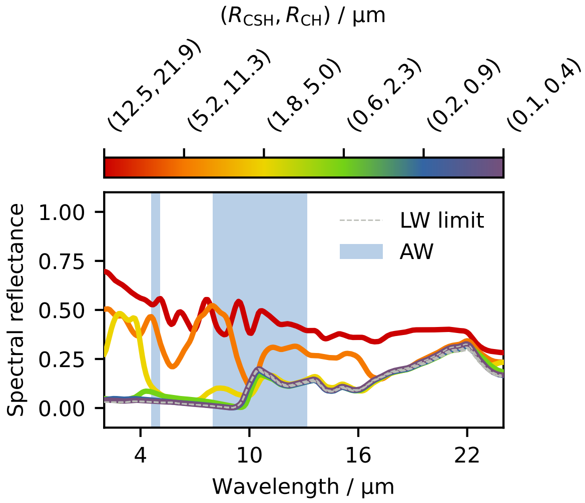

Figure S4: Spectral reflectance (angular-average) of the cement layer as a function of the mean particle (sub-domain) radii $(R_{\mathrm{CSH}}, R_{\mathrm{CH}})$. Related to **Figure 5**.

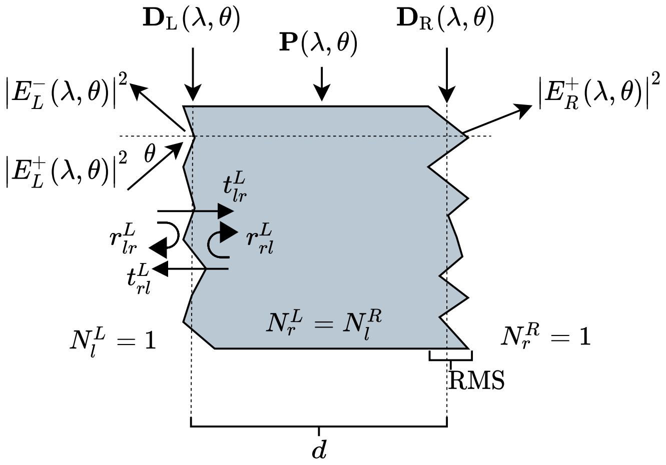

Figure S5: Depiction of the generalized transfer-matrix method applied to a single slab with rough surfaces and incoherent propagation. Related to **STAR Methods**. All the quantities used for the calculation of the macroscopic electromagnetic response (reflectance, transmittance and absorbance) are represented.

Table S1: Stoichiometry and conventional lattice constants of the studied C_3S, CSH **and** CH **structures.** Related to **STAR Methods**. The values in parenthesis are the experimental ones. a, b and c are the lengths of the conventional unit cell translation vectors, while α, β and γ are the angles between them.

	C_3S	CSH	CH
Composition	Ca_3SiO_5	$(CaO)_{254}(SiO_2)_{152}(H_2O)_{306}$	$Ca(OH)_2$
a/Å	12.36 (12.23)	26.08	3.55 (3.59)
b/Å	7.13 (7.03)	30.84	3.55 (3.59)
c/Å	9.57 (9.29)	25.88	4.94 (4.90)
$\alpha/^\circ$	90 (90)	90	90 (90)
$\beta/^\circ$	115.48 (116.31)	90	90 (90)
$\gamma/^\circ$	90 (90)	90	120 (120)
